# Intravenous zanamivir for influenza myocarditis and enteral malabsorption

**DOI:** 10.1186/s13054-018-2263-y

**Published:** 2018-12-04

**Authors:** Fritz-Patrick Jahns, Nawfel Ben-Hamouda, Matthias Kirsch, Aurélien Roumy, Lucas Liaudet

**Affiliations:** 1 Department of Intensive Care Medicine and Burns, Lausanne University Hospital Medical Center, CHUV, 1011 Lausanne, Switzerland; 2 Department of Cardiovascular Surgery, Lausanne University Hospital Medical Center, 1011 Lausanne, Switzerland

**Keywords:** Influenza, Myocarditis, Drug malabsorption, Extracorporeal life support

Acute myocarditis is an uncommon complication of influenza with a high mortality [1]. Early therapy with neuraminidase inhibitors (NI) is recommended in patients hospitalized for influenza, notably those with myocarditis. Oral oseltamivir is generally used as the first line NI therapy, whereas parenteral zanamivir and peramivir represent alternatives in selected patients who might not respond to oseltamivir, as may occur in conditions of gut failure and defective enteral drug absorption [2, 3]. We present two patients with influenza myocarditis complicated by enteral drug malabsorption, who received early intravenous zanamivir therapy with excellent clinical outcomes.

**Fig. 1:**
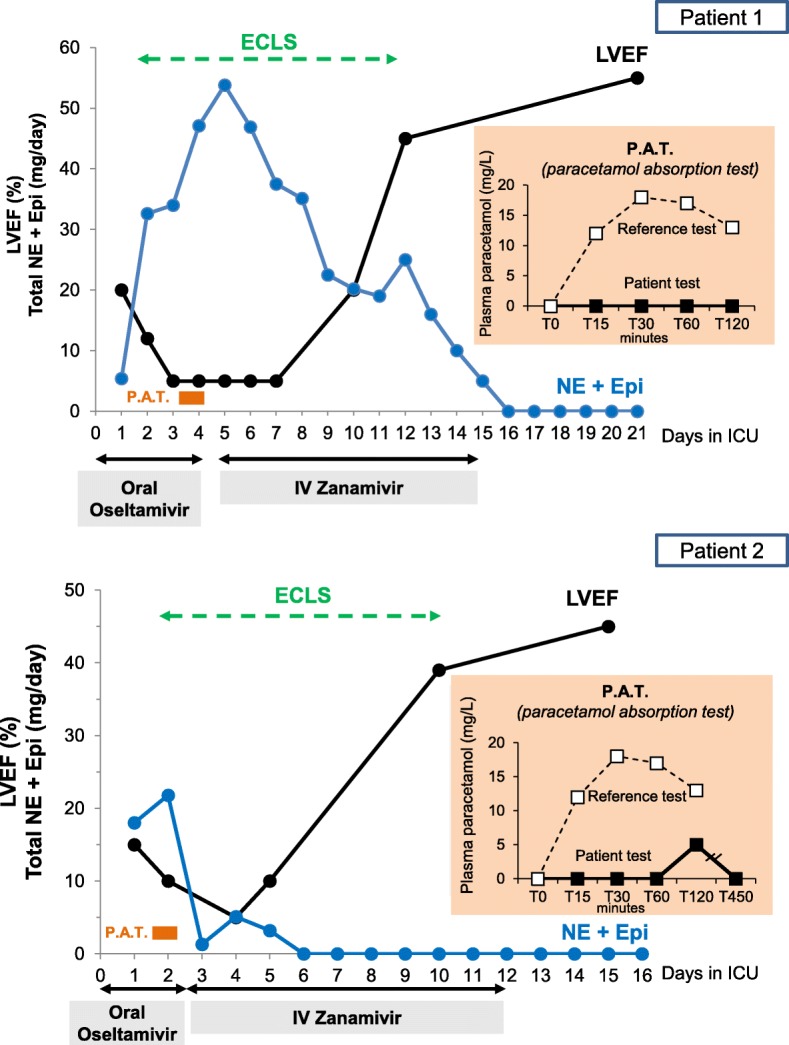
Time-course of LVEF, catecholamines, and ECLS in two patients with fulminant influenza myocarditis. LVEF rapidly increased, allowing weaning of norepinephrine (*NE*), epinephrine (*Epi*), and extracorporeal life support (*ECLS*), after switching from oral oseltamivir to intravenous (*IV*) zanamivir, following a paracetamol absorption test (*P.A.T.*) showing enteral drug malabsorption (*insert*). The reference values for the test were obtained from [4]

Patient 1 is a 49-year old woman, admitted with hypotension (71/47 mmHg), high hsTroponin T (up to 1570 ng/l), severe cardiac dysfunction (left ventricular ejection fraction (LVEF) of 20%), and positive naso-pharyngeal swabs for influenza B. Patient 2 is a 30-year old woman admitted with hypotension (82/54 mmHg), severe cardiac dysfunction (LVEF of 15%), elevated hsTroponin T (up to 3643 ng/l), and positive swabs for influenza A. Both patients received oral oseltamivir (300 mg daily) and intravenous catecholamines (Fig. 1). In both, extracorporeal life support (ECLS) was started at day 2 for refractory cardiogenic shock. Given clinical deterioration, enteral drug absorption was assessed at day 4 for patient 1 and day 2 for patient 2 by a paracetamol absorption test [4], which measures sequential plasma paracetamol levels after enteral loading (via nasogastric tube) with 1000 mg paracetamol. A marked impairment of enteral drug absorption was identified in both patients, leading to a switch from oral oseltamivir to parenteral zanamivir (patient 1, 600 mg daily, adapted to renal function, from days 5 to 15; patient 2, 1200 mg daily, from days 2 to 12). In the following days, catecholamines were steadily reduced, LVEF progressively improved, and ECLS was weaned (patient 1, day 12; patient 2, day 10). At ICU discharge, LVEF was 55% and 45%, respectively.

In these two patients with influenza myocarditis and refractory cardiogenic shock, the rapid identification of poor drug absorption using a simple paracetamol absorption test allowed the early introduction of parenteral zanamivir instead of oral oseltamivir, with excellent clinical outcomes. Since drug malabsorption is frequent in critically ill patients with circulatory shock [5], we propose that a paracetamol pharmacokinetic study be performed early in patients with influenza myocarditis and cardiogenic shock to avoid any delay in the administration of parenteral therapy if enteral drug malabsorption is demonstrated.
